# Author's Reply

**DOI:** 10.18502/jovr.v14i4.5477

**Published:** 2019-10-24

**Authors:** Hamid Sajjadi, Hossein Poorsalman

**Affiliations:** ^1^ San Jose Eye and Laser Medical Center, Cupertino, California, USA; ^2^ Department of Ophthalmology, Acacia Medical Center. Dubai, UAE; ^3^ Department of Ophthalmology, Red Crescent Hospital, Dubai, UAE

Sir,

Thank you for your interest in our article.^[1]^ You raised some questions about our case report; below please notice our answers:

1. As we mentioned in our article the mitochondrial mutation for Leber hereditary optic neuropathy (LHON) was not present in this case and the patient had been labeled as LHON by previous clinicians. Therefore, the diagnosis of LHON was completely presumptive.

The ICP that was measured in our case was 18 cmH2O in lateral decubitus position which used to be high before 2016; however, it has been challenged in recent articles that in children it can be up to 26 cmH2O. However, these articles also state that in children you have to interpret the ICP measurement in the context of clinical signs and symptoms, there in effect an lumbar puncture (LP) is not reliable in children. Our patient had some MRI signs of pseudotumor cerebri (PTC) including extensive fluid around the optic nerve and hygroma. Hygromas are normal in older than 60 years olds but not in a child. There are also ample reports of low tension PTCs in the literature.^[2,3]^ Normal IOP does not exclude PTC and what makes our report unique is the dramatic response of vision and stereopsis to the ICP lowering treatment.

2. As optic atrophy is an ultimate sign of every optic neuropathy, finding a way in elucidating the definite cause of it is really important. There are lots of cases of optic atrophy with unknown cause and there should be a way to explain their origin. Our previous article was an effort to open this discussion and was based on many neuro-ophthalmology cases that we have seen in these years. Our diagnosis was not merely based on the optic nerve head (ONH) feature of our patient, but was also dependent on the MRI findings and the dramatic response to acetazolamide therapy. As explained, we have reported 164 cases of LP proven PTC without visible papilledema.^[3]^ Many of these cases presented with optic atrophy and no papilledema but did have high ICP on LP.

3. We think our pictures have the best possible quality with Topcon OCT device and no other machine would produce a better ganglion cell layer (GCL) analysis. The first picture was taken by a Topcon 2000 machine and the later ones were done at different center by the most recent software; therefore, they look different. However, the presence of central scotoma is obvious in the original GCL analysis which opened up as the patient's central vision improved. In his last visit (4 months ago), his BCVA was 20/20 in each eye.

4. It is mentioned in our article that the mitochondrial mutation for LHON was not present in this patient. There were neither 14484 nor any other mutations in our case. Therefore, the theory of visual recovery secondary to this mutation cannot be correct.

5. Although spontaneous stopping of damage has been reported in LHON, an improvement from 20/200 to 20/20 has never been reported.
